# Prognostic and clinicopathological significance of Systemic Immune-Inflammation Index in cancer patients receiving immune checkpoint inhibitors: a meta-analysis

**DOI:** 10.1080/07853890.2023.2181983

**Published:** 2023-03-09

**Authors:** Yan Wang, Qunqin Ni

**Affiliations:** a Clinical Laboratory, Huzhou Central Hospital, Affiliated Central Hospital of Huzhou University, Huzhou, Zhejiang, China; b Clinical Laboratory, Traditional Chinese Medical Hospital of Huzhou Affiliated to Zhejiang Chinese Medical University, Huzhou, Zhejiang, China

**Keywords:** immune checkpoint inhibitors, meta-analysis, SII, prognosis, evidence-based medicine

## Abstract

**Background:**

Among malignant neoplasm patients taking immune checkpoint inhibitors (ICIs), it remains unknown how the systemic immune-inflammation index (SII) affects their clinical prognosis. We therefore performed the present meta-analysis by collecting the most recent data, so that SII’s prognostic value among ICI-receiving carcinoma patients could be fully clarified.

**Methods:**

For the prognostic significance evaluation of SII in ICI-receiving carcinoma patients, the combined hazard ratios (HRs) and 95% confidence intervals (CIs) were estimated.

**Results:**

The number of studies enrolled in the present meta-analysis totaled 17, where 1,990 patients were involved. Among the ICI-treated carcinoma patients, a high SII was linked significantly to inferior overall survival (OS) (HR = 2.62, 95% CI = 1.76–3.90), as well as progression-free survival (PFS) (HR = 2.09, 95% CI = 1.48–2.95) (*p* both <.001). Contrastively, SII was linked insignificantly to the age (OR = 1.08, 95% CI = 0.39–2.98, *p* = .881), gender (OR = 1.01, 95% CI = 0.59–1.73, *p* = .959), lymph node (LN) metastasis (OR = 1.41, 95% CI = 0.92–2.17, *p* = .117), or metastatic site quantity (OR = 1.49, 95% CI = 0.90–2.46, *p* = .119).

**Conclusion:**

There are prominent associations of elevated SII with the poor survival outcomes (both short- and long-terms) among the ICIreceiving carcinoma patients. SII has potential as a reliable and cheap prognostic biomarker in the clinic for carcinoma patients receiving ICIs.

## Key Messages

- The current meta-analysis represents the first attempt to explore SII’s prognostic efficiency among carcinoma patients treated with ICIs.
- There are prominent associations of elevated SII with the poor survival outcomes (both short- and long-terms) among the ICI-receiving carcinoma patients.
- SII may serve as a reliable and cheap prognostic biomarker in the clinical settings.

## Introduction

Being the leading cause of death, cancer crucially impedes prolongation of the anticipated life span on a global scale [1]. According to the GLOBOCAN 2020 estimates in 185 countries around the world, there were 19,292,789 new cancer cases, as well as 9,958,133 cases died due to cancer in 2020 [1]. Regarding treatment for patients with cancer, surgery, chemotherapy, and radiotherapy are mainstay in clinical practice. In recent years, immune checkpoint inhibitors (ICIs) have been more and more commonly applied and have dramatically improved survival in various malignancies [2]. ICIs contain therapeutic antibodies that disrupt negative immune regulatory checkpoints and therefore enhance antitumour immune responses [3]. ICIs such antibodies targeting programmed cell death 1 (PD-1), cytotoxic T lymphocyte-associated protein 4 (CTLA-4) and PD1 ligand 1 (PD-L1) have showed promising therapeutic effect and have been applied for many cancer types in the clinic [4,5]. Novel and reliable prognostic biomarker identification is crucial to attaining individualized therapy among carcinoma patients receiving ICIs in clinical settings [6].

Growing evidence in recent years has demonstrated the heavy implications of inflammatory and immunoresponses in the cancer development [7]. For the carcinoma patients undergoing ICIs, their parameters derived from blood test have been explored as valid biomarkers for outcomes [8]. The haematological markers include the neutrophil–lymphocyte ratio (NLR) [8], the platelet–lymphocyte ratio (PLR) [9], the modified Glasgow prognostic score (mGPS) [10], as well as the systemic inflammation index (SII) [11]. Computational equation for SII is: SII  =  platelet × neutrophil/lymphocyte [12]. SII is presented as a combination of NLR and PLR. SII’s prognostic effects have been priorly reported for many tumours [13,14]. Growing studies also explored its prognostic role in a variety of ICI-receiving carcinoma patients; however, the results remained controversial [11,15–30]. For instance, some investigators regarded SII as a significant marker to suggest oncological results in cancer patients receiving ICIs [19,20], whereas some other investigators found the association was non-significant [15,30]. Hence, for a complete prognostic value assessment on SII among ICI-receiving carcinoma patients, we collected the most recent data and performed this meta-analysis. Additionally, we examined how SII was associated with the clinicopathological traits in cancer patients receiving ICIs as well.

## Materials and methods

### Study guideline

The present meta-analysis was carried out as per the Preferred Reporting Items for Systematic Reviews and Meta-Analyses (PRISMA) guideline [31].

### Ethics statement

Since all the data in the present meta-analysis were retrieved from public databases, neither the institutional review board nor the ethics committee was required.

### Search strategy

Literature was retrieved in a systematic and holistic manner by utilizing online databases like Web of Science, PubMed, Cochrane Library and Embase between the dates of inception and 1 January 2023. We registered this meta-analysis protocol in INPLASY. The registration number is INPLASY202310018.The search terms shown below were used: (SII or Systemic Immune-Inflammation Index) and (immune check point inhibitor or PD-L1 or PD-1 or avelumab or CTLA-4 or durvalumab or nivolumab or ipilimumab or atezolizumab or pembrolizumab or camrelizumab or tislelizumab or toripalimab or penpulimab or immune checkpoint blockade or immunotherapy). Only the articles published in English were retrieved. Possible inclusions were recognized also by prudently checking the references in qualified researches, as well as relevant reviews.

### Inclusion and exclusion criteria

The inclusion criteria were as follows: (1) patients were pathologically diagnosed with cancer and treated with ICIs; (2) available data of pretreatment SII; (3) studies investigating the relationship between SII and prognosis of patients with cancer undergoing ICIs; (4) a cut-off value was identified to stratify patients as high/low SII groups; (5) any survival outcomes were reported including overall survival (OS), progression-free survival (PFS), recurrence-free survival (RFS) etc., (6) studies published in English language. The exclusion criteria were: (1) reviews, letters, meeting abstracts, and case reports; (2) studies did not provide survival data; (3) duplicate studies and (4) non-human studies.

### Data extraction and quality assessment

Relevant researches were collected by two independent researchers (Y.W. and Q.N.) through retrieval and scanning of identified titles and abstracts. All disagreements were addressed through negotiation until reaching consensus. Information extracted from qualified researches included: first author’s name, country, sample size, publication year, study design, age, gender, cancer type, study period, ICIs type, tumour-node-metastasis (TNM) stage, study centre, survival outcomes, cut-off value of SII, follow-up, hazard ratio (HR), survival assessment, as well as 95% confidence interval (CI). Pooling of HRs and 95% CIs was accomplished from multivariate assessments if provided. Otherwise, univariate assessments were the source of HRs and 95% CIs. The enrolled studies were subjected to quality evaluation *via* the Newcastle–Ottawa Scale (NOS) [32]. This scale assesses the research quality regarding selection, comparability and prognosis. Studies scoring ≥6 on this nine-point NOS are considered to have high quality.

### Statistical analysis

SII’s prognostic significance among the ICI-receiving carcinoma patients was evaluated by estimating the combined HRs and 95% CIs. Cochran’s Q test was employed to assess the inter-study heterogeneity by utilizing I^2^ statistics. In the case of insignificant heterogeneity (*p* > .05 for χ^2^ test or I^2^< 50%), a fixed-effect model was adopted. Otherwise, a random-effects model was selected. Heterogeneity source was identified by conducting subgroup analysis stratified by diverse variables. How SII was associated with the clinicopathological traits of cancer patients using ICIs was assessed by pooling odds ratios (ORs) and 95%CIs. Possible publication bias was evaluated through Begg’s test. Stata 12.0 (College Station, TX) was utilized to analyse the entire data. *p* < .05 was regarded as statistically significant.

## Results

### Search results

**Figure 1.**
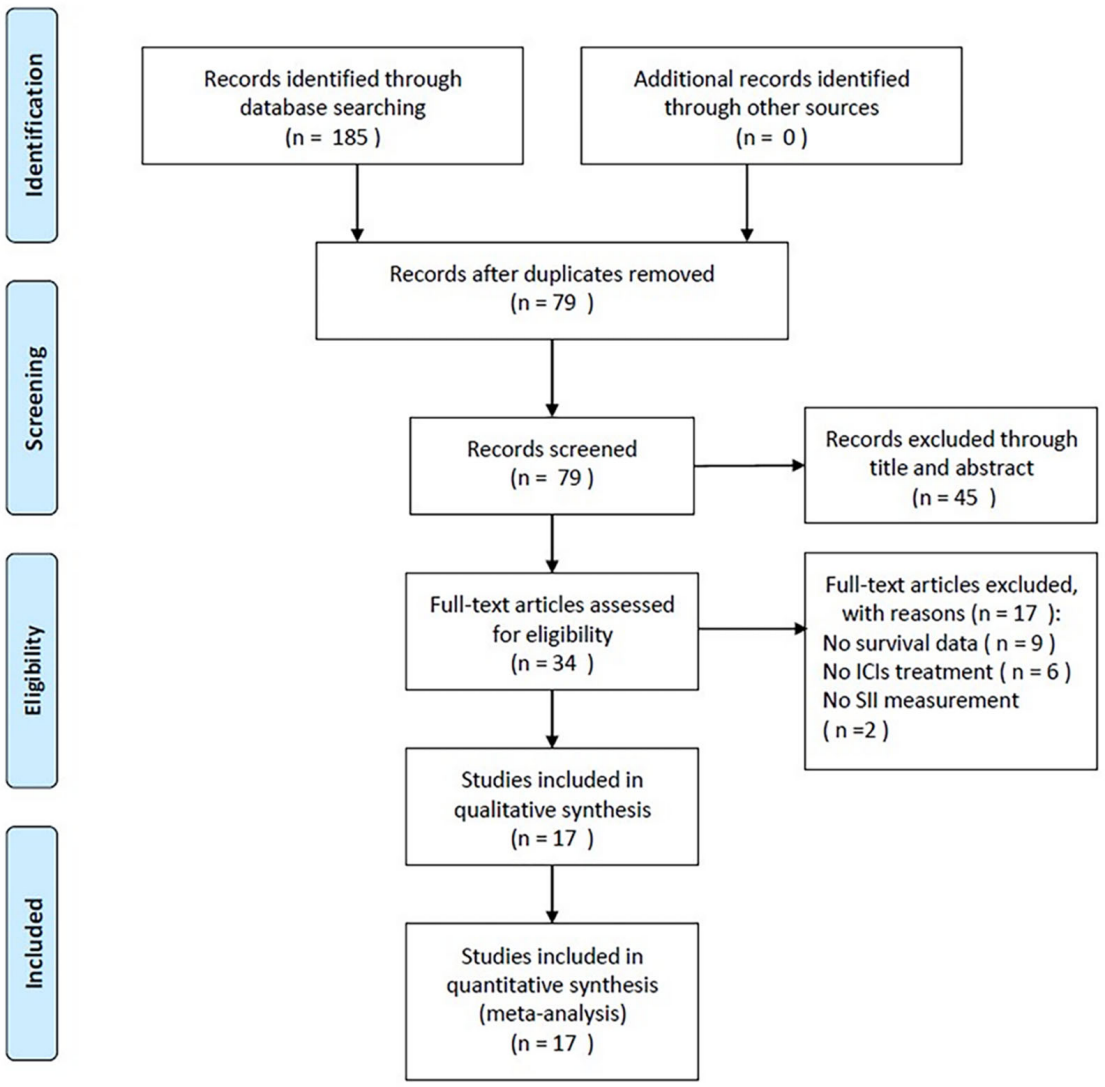
Flowchart representation of the steps of the literature search and selection in this meta-analysis.

We identified 185 publications through the initial literature retrieval, as displayed in Figure 1. After removal of duplicates, 79 studies remained for abstract and title scanning. Then 45 records were eliminated by title and abstract and 34 studies were assessed further *via* full-text reading. Thereafter, we eliminated 17 studies due to no survival data (*n*  =  9), no ICIs treatment (*n*  =  6), and no information on SII provided (*n*  =  2). Finally, 17 studies were enrolled in total in the present meta-analysis, where 1990 patients were involved [11,15–30]. Figure 1 illustrates the flowchart for screening the studies.

### Characteristics of included studies

Table 1 details the baseline features of the enrolled studies [11,15–30], T whose publication years ranged from 2019 to 2022. Among them, nine studies were carried out in China [15,16,18,21,25–27,29,30], three studies were carried out in Italy [11,17,20], two studies were performed in Germany [23,28], and one each in Japan [22], Korea [24], and Turkey [19], respectively. English was the language of publication for all included studies. The scope of sample sizes was 29–313, and the median value was 106. Four studies were prospective clinical trials [11,17,20,23] and 13 studies were of retrospective design [15,16,18,19,21,22,24–30]. Three studies recruited non-small cell lung cancer (NSCLC) patients [11,23,25], three studies enrolled renal cell carcinoma (RCC) patients [17,22,28], two studies included patients with oesophageal cancer [15,29], two studies enrolled patients with gastric cancer [16,26], and one each study recruited patients with biliary tract cancer (BTC) [18], hepatocellular carcinoma (HCC) [21], melanoma [24], pancreatic cancer [27], small cell lung cancer (SCLC) [30], urothelial carcinoma [20], and various cancers [19], respectively. Nine studies included patients with TNM stage IV [11,17–19,22–24,26,28], seven studies enrolled patients with stage III-IV [15,16,20,21,25,27,29], and one study enrolled stage I-IV SCLC patients [30]. Regarding ICIs type, ten studies used anti-PD-1 antibodies [11,15,17,18,21,23–26,29], two studies applied anti-PD-1/anti-PD-L1 antibodies [16,30], two studies used anti-PD-1 plus anti-CTLA-4 antibodies [22,28], two studies selected anti-PD-1/PD-L1 + anti-CTLA-4 antibodies [19,27] and one study applied anti-PD-L1 antibodies [20]. The SII threshold scope was 197.2–1375, with a median of 829. Thirteen studies [11,15–21,24–28] reported SII’s prognostic significance to OS, while 14 studies [15,16,18,20–30] presented the SII–PFS correlation among the ICI-treated carcinoma patients. For the enrolled studies, the NOS score scope was 7–9, with a median of 8, revealing high quality of the entire studies. Table 2 lists the particular NOS scores.

**Table 1.** Basic characteristics of included studies in the current meta-analysis.

Study	Year	Country	Sample size	Study design	Gender (M/F)	Cancer type	Age Median (range) year	Study centre	TNM stage	ICIs type	Study period	SII cut-off value	Outcome	Follow-up Median (range) (months)	Survival analysis	NOS score
Bauckneht, M. et al. [11]	2021	Italy	45	Prospective	29/16	NSCLC	70.6(50.3–81.5)	Multicentre	IV	Nivolumab	2015–2020	197.2	OS	9.43	Univariate	8
Chang, L. et al. [15]	2022	China	69	Retrospective	67/2	Esophageal cancer	60(44–78)	Single centre	III–IV	Camrelizumab/ Sintilimab/ Toripalimab	2017–2020	837	OS, PFS	1–30	Multivariate	8
Chen, Y. et al. [16]	2021	China	139	Retrospective	103/36	Gastric cancer	60	Single centre	III–IV	Anti-PD-1/PD-L1	2015–2019	665.3	OS, PFS	23.8	Multivariate	7
De Giorgi, U. et al. [17]	2019	Italy	313	Prospective	235/78	RCC	65(40–84)	Multicentre	IV	Nivolumab	2015–2016	1375	OS	1–24	Multivariate	9
Du, F. et al. [18]	2021	China	60	Retrospective	41/19	BTC	61(28–83)	Multicentre	IV	Anti-PD-1	2016–2019	720	OS, PFS	1–28	Univariate	8
Ekinci, F. et al. [19]	2022	Turkey	168	Retrospective	119/49	Mixed	<65 y: 101 ≥65 y: 67	Multicentre	IV	Nivolumab/ Atezolizumab/ Pembrolizumab/ Ipilimumab	2016–2020	1156	OS	91	Multivariate	8
Fornarini, G. et al. [20]	2021	Italy	267	Prospective	221/46	Urothelial carcinoma	69	Multicentre	III–IV	Atezolizumab	2016–2020	1375	OS, PFS	9.5	Multivariate	9
Huang, R. et al. [21]	2022	China	110	Retrospective	100/10	HCC	54.5(31–84)	Single centre	III–IV	Anti-PD-1	2019–2020	970	OS, PFS	1–20	Multivariate	7
Iinuma, K. et al. [22]	2021	Japan	43	Retrospective	31/12	RCC	69	Multicentre	IV	Nivolumab + Ipilimumab	2018–2021	561.7	PFS	14	Univariate	8
Kauffmann et al. [23]	2021	Germany	29	Prospective	24/5	NSCLC	64.1	Single centre	IV	Nivolumab/ Pembrolizumab	2016–2019	1217	PFS	1–46	Univariate	8
Lee, J. H. et al. [24]	2022	Korea	266	Retrospective	135/131	Melanoma	60	Single centre	IV	Nivolumab/ Pembrolizumab	2015–2021	850	OS, PFS	13.9	Multivariate	7
Liu, J. et al. [25]	2019	China	44	Retrospective	33/11	NSCLC	60(43–74)	Single centre	III–IV	Nivolumab	2016–2018	603.5	OS, PFS	6.9	Multivariate	8
Qu, Z. et al. [26]	2022	China	106	Retrospective	72/34	Gastric cancer	>65 y: 27 ≤65 y: 77	Single centre	IV	Anti-PD-1	2019–2021	1140.9	OS, PFS	17.5	Univariate	8
Shang, J. et al. [27]	2021	China	122	Retrospective	87/35	Pancreatic cancer	56(33–85)	Single centre	III–IV	Nivolumab/ Pembrolizumab/ Atezolimab/ Ipilimumab/ Sintilimab	2015–2019	566	OS, PFS	1–80	Multivariate	7
Stühler, V. et al. [28]	2022	Germany	49	Retrospective	35/14	RCC	64.6(39.9–83.5)	Single centre	IV	Nivolumab + Ipilimumab	2012–2018	788	OS, PFS	9.53(0.33–45.9)	Multivariate	8
Wu, X. et al. [29]	2021	China	119	Retrospective	102/17	Esophageal cancer	61(42–78)	Single centre	III–IV	Anti-PD-1	2018–2020	829	PFS	1–21	Univariate	7
Xiong, Q. et al. [30]	2021	China	41	Retrospective	36/5	SCLC	61(42–80)	Single centre	I–IV	Anti-PD-1/PD-L1	2015–2018	730	PFS	1–8	Univariate	8

*Note:* NSCLC: non-small cell lung cancer; RCC: renal cell carcinoma; BTC: biliary tract cancer; HCC: hepatocellular carcinoma; SCLC: small cell lung cancer; OS: overall survival; PFS: progression-free survival; NOS: Newcastle-Ottawa Scale; SII: Systemic Immune-Inflammation Index.

**Table 2.** The details of NOS scale for included studies in this meta-analysis.

		Selection（0–4 point）				Comparability (0–2 point)	Outcome (0–3 point)			
Study	Year	Representativeness of the exposed cohort	Selection of the non exposed cohort	Ascertainment of exposure	Demonstration that outcome of interest was not present at start of study	Comparability of cohorts on the basis of the design or analysis	Assessment of outcome	Was follow-up long enough for outcomes to occur	Adequacy of follow up of cohorts	Total score
Bauckneht, M. et al. [11]	2021	★	★	★	★	★★	★	⋆	★	8
Chang, L. et al. [15]	2022	★	★	⋆	★	★★	★	★	★	8
Chen, Y. et al. [16]	2021	★	★	★	★	★⋆	★	⋆	★	7
De Giorgi, U. et al. [17]	2019	★	★	★	★	★★	★	★	★	9
Du, F. et al. [18]	2021	★	★	⋆	★	★★	★	★	★	8
Ekinci, F. et al. [19]	2022	★	★	★	★	★⋆	★	★	★	8
Fornarini, G. et al. [20]	2021	★	★	★	★	★★	★	★	★	9
Huang, R. et al. [21]	2022	★	★	⋆	★	★⋆	★	★	★	7
Iinuma, K. et al. [22]	2021	★	★	★	★	★⋆	★	★	★	8
Kauffmann et al. [23]	2021	★	★	★	★	★★	★	⋆	★	8
Lee, J. H. et al. [24]	2022	★	★	★	★	★★	★	★	⋆	7
Liu, J. et al. [25]	2019	★	★	★	★	★★	★	⋆	★	8
Qu, Z. et al. [26]	2022	★	★	★	★	★⋆	★	★	★	8
Shang, J. et al. [27]	2021	★	★	⋆	★	★★	★	⋆	★	7
Stühler, V. et al. [28]	2022	★	★	★	★	★⋆	★	★	★	8
Wu, X. et al. [29]	2021	★	★	★	★	★⋆	★	★	⋆	7
Xiong, Q. et al. [30]	2021	★	★	⋆	★	★★	★	★	★	8

*Note:* NOS: Newcastle-Ottawa Scale. A ★ represents 1 point, a ⋆ represents 0 point.

### Prognostic role of SII for OS

**Figure 2.**
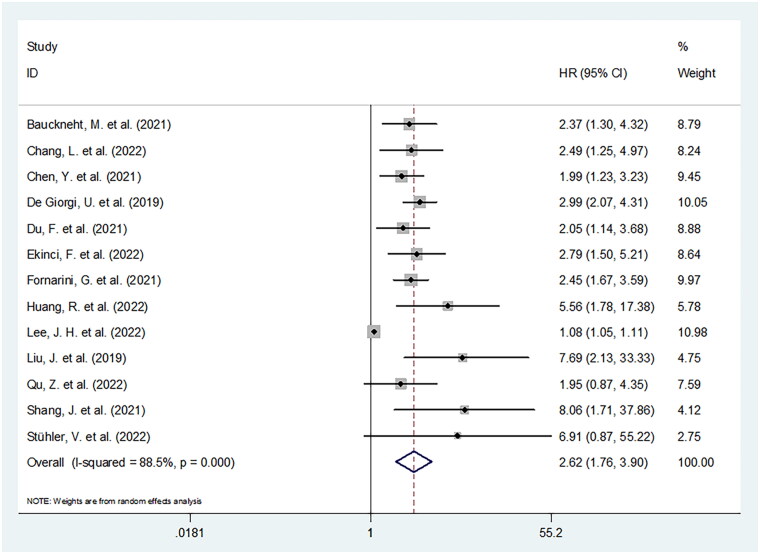
Forest plot of HR for the relationship between SII and overall survival in cancer patients receiving ICIs.

SII was shown prognostically significant for carcinoma patients undergoing ICIs in 13 studies involving 1758 patients in total [11,15–21,24–28]. Significant heterogeneity (I^2^ = 88.5%, *p* < .001) was detected, and we employed a random-effects model. The pooled HR was 2.62, whereas 95% CIs ranged 1.76–3.90 (*p* < .001) (Figure 2, Table 3), implying prominent correlation of high SII with inferior OS. According to the subgroup assessments stratified by multiple factors, SII’s prognostic significance in OS was unaffected by study design, sample size, study centre, TNM stage, cut-off value, geographical region, cancer type, and survival analysis type in cancer patients treated with ICIs (all *p* < .05) (Table 3).

**Figure 3.**
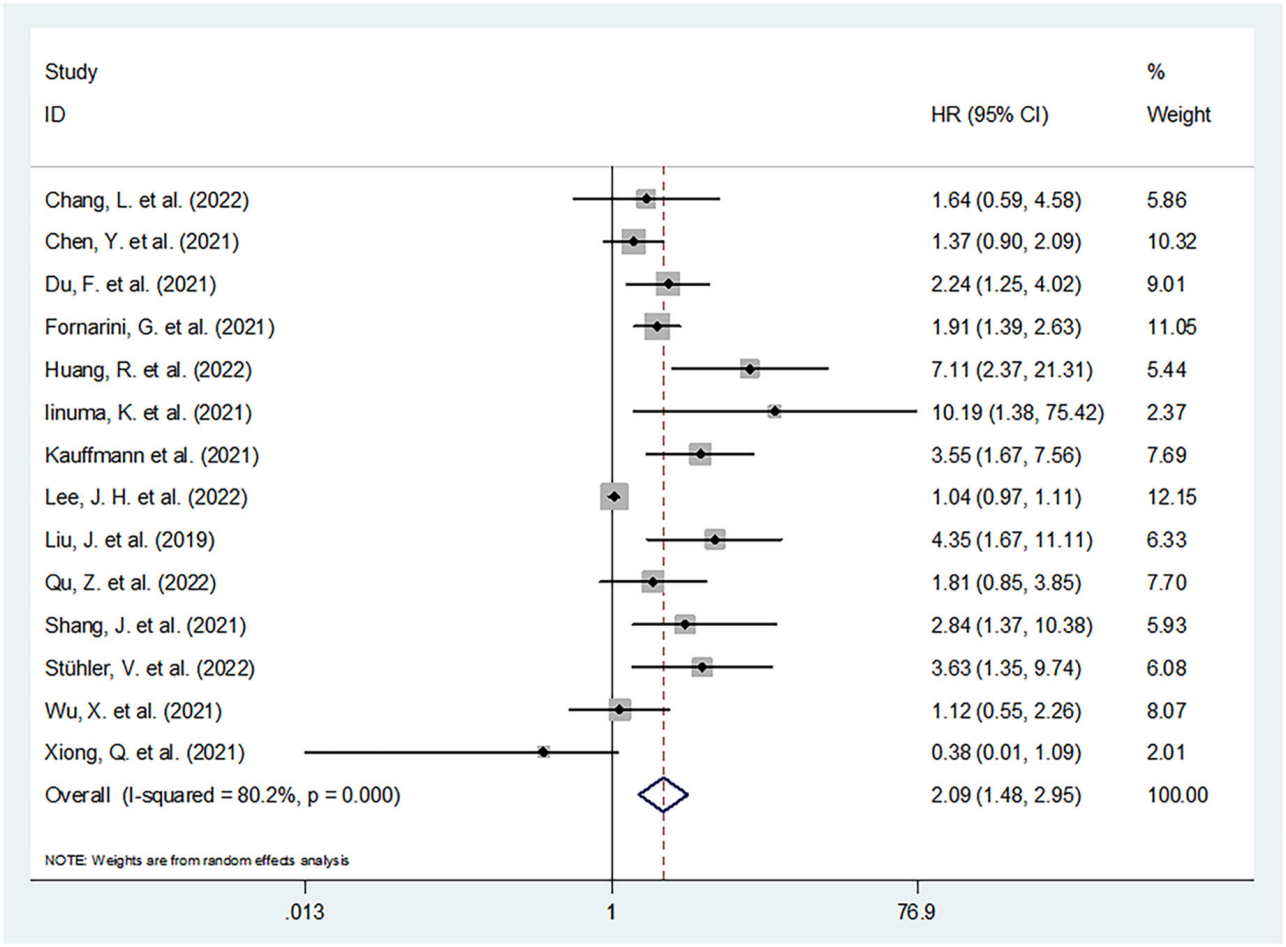
Forest plot of HR for the association between SII and progression-free survival in cancer patients receiving ICIs.

**Table 3.** The prognostic value of SII for OS in cancer patients treated with ICIs.

Subgroups	No. of studies	No. of patients	Effects model	HR (95%CI)	*p*	Heterogeneity I^2^ (%) Ph	
Overall	13	1,758	Random	2.62(1.76–3.90)	<.001	88.5	<0.001
Study design							
Prospective	3	625	Fixed	2.66(2.09–3.39)	<.001	0	0.702
Retrospective	10	1,133	Random	2.62(1.64–4.18)	<.001	82.7	<0.001
Sample size							
<100	5	267	Fixed	2.53(1.80–3.56)	<.001	0	0.413
≥100	8	1,491	Random	2.46(1.50–4.03)	<.001	91.0	<0.001
Study centre							
Single centre	8	905	Random	2.76(1.56–4.86)	<.001	82.0	<0.001
Multicentre	5	853	Fixed	2.58(2.09–3.19)	<.001	0	0.841
TNM stage							
III–IV	6	751	Fixed	2.60(2.01–3.36)	<.001	32.8	0.190
IV	7	1007	Random	2.18(1.30–3.65)	.003	88.9	<0.001
ICIs type							
Anti-PD-1	8	1117	Random	2.45(1.47–4.09)	.001	89.0	<0.001
Anti-PD-1/PD-L1	1	41	–	1.99(1.23–3.22)	.005	–	–
Anti-PD-1/PD-L1 + Anti-CTLA-4	2	290	Fixed	3.24(1.81–5.78)	<.001	35.4	0.213
Anti-PD-1 + Anti-CTLA-4	1	43	–	6.91(0.87–55.05)	.068	–	–
Anti-PD-L1	1	267	–	2.45(1.67–3.59)	<.001	–	–
Cut-off value of SII							
<800	6	459	Fixed	2.42(1.79–3.26)	<.001	28.1	0.224
≥800	7	1299	Random	2.35(1.39–3.96)	.001	91.5	<0.001
Country							
Asian	9	1084	Random	2.51(1.57–4.02)	<.001	83.7	<0.001
Non-Asian	4	674	Fixed	2.69(2.12–3.43)	<.001	0	0.680
Survival analysis							
Univariate	3	211	Fixed	2.15(1.48–3.11)	<.001	0	0.910
Multivariate	10	1547	Random	2.85(1.75–4.64)	<.001	90.2	<0.001
Cancer type							
NSCLC	2	89	Random	3.61(1.20–10.87)	.023	57.6	0.125
Esophageal cancer	1	69	–	2.49(1.24–4.97)	.010	–	–
Gastric cancer	2	245	Fixed	1.98(1.31–2.99)	.001	0	0.964
RCC	2	362	Fixed	3.07(2.14–4.40)	<.001	0	0.436
BTC	1	60	–	2.05(1.14–3.68)	.016	–	–
Mixed	1	168	–	2.79(1.49–5.21)	.001	–	–
Urothelial carcinoma	1	267	–	2.45(1.67–3.59)	<.001	–	–
HCC	1	110	–	5.56(1.78–17.38)	.003	–	–
Melanoma	1	266	–	1.08(1.05–1.11)	<.001	–	–
Pancreatic cancer	1	122	–	8.06(1.71–37.93)	.008	–	–

*Note:* SII: Systemic Immune-Inflammation Index; OS: overall survival; ICIs: immune checkpoint inhibitors; NSCLC: non-small cell lung cancer; RCC: renal cell carcinoma; BTC: biliary tract cancer; HCC: hepatocellular carcinoma.

### Prognostic impact of SII on PFS

SII produced a prognostic influence on PFS in 14 studies comprising 1464 patients [15,16,18,20–30]. Given the significant inter-study heterogeneity (I^2^ = 80.2%, *p* < .001), we adopted a random-effects model. As displayed in Figure 3 and Table 4, the pooled HR was 2.09, while 95% CIs ranged from 1.48 to 2.95 (*p* < .001). As revealed by subgroup assessments, SII’s prognostic impact was kept stable across various subgroups of sample size, research design, centre, geographical location, threshold, as well as type of survival analysis (all *p* < .05) (Table 4).

**Figure 4.**
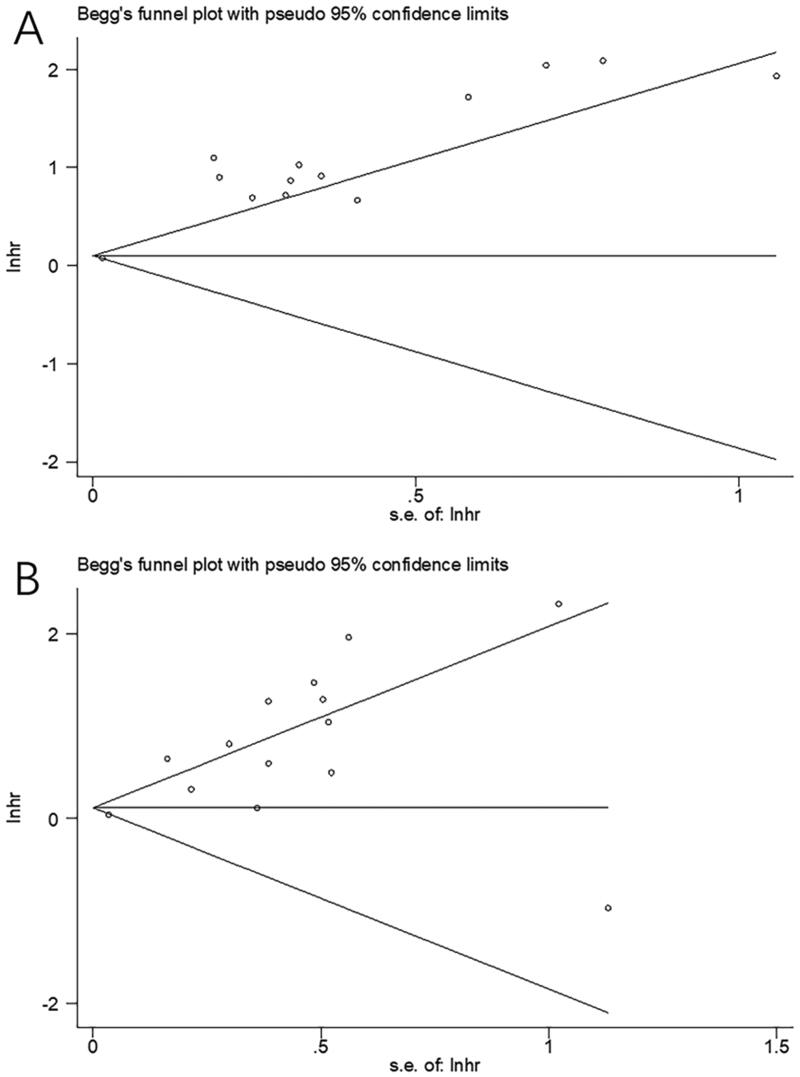
Results of the analysis of publication bias. (A) OS, Begg’s test, *p* = .583; (B) PFS, Begg’s test, *p* = .956.

**Table 4.** The prognostic value of SII for PFS in cancer patients treated with ICIs.

Subgroups	No. of studies	No. of patients	Effects model	HR (95%CI)	*p*	Heterogeneity I^2^ (%) Ph	
Overall	14	1464	Random	2.09(1.48–2.95)	<.001	80.2	<0.001
Study design							
Prospective	2	296	Random	2.36(1.33–4.20)	.004	54.6	0.138
Retrospective	12	1168	Random	2.02(1.37–2.98)	<.001	75.7	<0.001
Sample size							
<100	7	335	Fixed	2.78(1.96–3.93)	<.001	23.0	0.254
≥100	7	1129	Random	1.68(1.15–2.44)	.007	80.8	<0.001
Study centre							
Single centre	11	1094	Random	2.02(21.34–3.03)	.001	77.4	<0.001
Multicentre	3	370	Fixed	2.04(1.55–2.70)	<.001	27.0	0.254
TNM stage							
III–IV	7	870	Random	2.06(1.40–3.03)	<.001	55.8	0.035
IV	6	553	Random	2.32(1.25–4.30)	.007	82.8	<0.001
I–IV	1	41	–	0.38(0.04–3.48)	.392	–	–
ICIs type							
Anti-PD-1	8	983	Random	2.15(1.30–3.55)	.003	82.0	<0.001
Anti-PD-1/PD-L1	2		Fixed	1.31(0.87–1.96)	.201	19.6	0.265
Anti-PD-1/PD-L1 + Anti-CTLA-4	1	122	–	2.84(1.03–7.82)	.043	–	–
Anti-PD-1 + Anti-CTLA-4	2	92	Fixed	4.44(1.83–10.78)	.001	0	0.365
Anti-PD-L1	1	267	–	1.91(1.39–2.63)	<.001	–	–
Cut-off value of SII							
<800	7	498	Random	2.40(1.47–3.93)	<.001	51.5	0.054
≥800	7	966	Random	1.87(1.20–2.93)	.006	83.7	<0.001
Country							
Asian	11	1119	Random	1.92(1.30–2.83)	.001	74.8	<0.001
Non-Asian	3	345	Fixed	2.19(1.66–2.91)	<.001	39.2	0.193
Survival analysis							
Univariate	6	398	Fixed	2.01(1.44–2.81)	<.001	48.7	0.083
Multivariate	8	1066	Random	2.13(1.38–3.28)	.001	84.1	<0.001
Cancer type							
NSCLC	2	73	Fixed	3.84(2.13–6.93)	<.001	0	0.744
Esophageal cancer	2	188	Fixed	1.27(0.71–2.26)	.425	0	0.545
Gastric cancer	2	245	Fixed	1.46(1.01–2.12)	.042	0	0.525
RCC	2	92	Fixed	4.44(1.83–10.78)	.001	0	0.365
BTC	1	60	–	2.24(1.25–4.02)	.007	–	–
Urothelial carcinoma	1	267	–	1.91(1.39–2.63)	<.001	–	–
HCC	1	110	–	7.11(2.37–21.31)	<.001	–	–
Melanoma	1	266	–	1.04(0.97–1.11)	.254	–	–
Pancreatic cancer	1	122	–	2.84(1.03–7.82)	.043	–	–
SCLC	1	41	–	0.38(0.04–3.48)	.392	–	–

*Note:* SII: Systemic Immune-Inflammation Index; PFS: progression-free survival; ICIs: immune checkpoint inhibitors; NSCLC: non-small cell lung cancer; RCC: renal cell carcinoma; BTC: biliary tract cancer; HCC: hepatocellular carcinoma; SCLC: small cell lung cancer.

### Association between SII and clinicopathological features

Four studies including 636 patients [17,19,26,28] reported the associations of SII with the clinicopathological traits for carcinoma patients receiving ICIs. As shown in Table 5, SII was linked insignificantly to the age (OR  = 1.08, 95% CI  = 0.39–2.98, *p*  =  .881), the gender (OR  = 1.01, 95% CI  = 0.59–1.73, *p*  =  .959), the lymph node (LN) metastasis (OR  = 1.41, 95% CI  = 0.92–2.17, *p*  =  .117), or the metastatic site quantity (OR  = 1.49, 95% CI  = 0.90–2.46, *p*  =  .119).

**Table 5.** The correlation between SII and clinicopathological variables in cancer patients undergoing ICIs treatment.

Variables	No. of studies	No. of patients	Effects model	OR (95%CI)	*p*	Heterogeneity I^2^ (%) Ph	
Gender (male vs female)	3	323	Fixed	1.01(0.59–1.73)	.959	0	0.529
Age (years) (≥65 vs <65)	2	274	Random	1.08(0.39–2.98)	.881	57.4	0.126
No. of metastasis sites (≥3 vs <3)	3	323	Fixed	1.49(0.90–2.46)	.119	46.8	0.152
LN metastasis (presence vs absence)	3	468	Fixed	1.41(0.92–2.17)	.117	0	0.747

### Publication bias

Possible publication bias was assessed by employing Begg’s test combined with funnel plot test. As shown in Figure 4, the funnel plots for OS and PFS were not evidently asymmetric in shape. With the Begg’s test, the *p* values for OS and PFS were .583 and .956, respectively. Thus, insignificant bias was present in our meta-analysis.

## Discussion

The previous works have shown that SII was prognostically significant among carcinoma patients receiving ICIs, whereas the results were inconsistent. In the present meta-analysis, data were extracted from 17 studies involving 1990 patients for quantitatively analysing the prognostic role of SII for OS and PFS. Our results demonstrated that elevated SII was a significant biomarker of outcome for both OS and PFS among the ICI-receiving carcinoma patients. Besides, SII exhibited a reliable prognostic efficiency, which was unaffected in diverse subgroups. However, SII was found associated significantly with age, gender, number of metastasis sites, or LN metastasis in our meta-analysis. Assessment of publication bias revealed the reliability of the results. In summary, our meta-analysis implied the significance and validity of SII as a biomarker of outcome for the ICI-treated carcinoma patients. Those cancer patients treated with ICIs who have high pretreatment SII may experience high risk of disease progression and should be cautiously managed.

The SII was derived as the formula: SII = (platelet quantity * neutrophil quantity)/lymphocyte quantity. Accordingly, a high SII would be indicative of high platelet and neutrophil quantities, and/or low quantity of lymphocytes. For the ICI-treated carcinoma patients, it is possible to explain the mechanisms of high SII with inferior survival prognosis in the following aspects: first, neutrophils can promote cancer development through directly interacting with tumour cells or indirectly remodelling the tumour microenvironment [33]. Growth and metastasis of tumours can also be promoted by neutrophils through the proinflammatory cytokine and chemokine discharge in tumour microenvironment [34]. Second, platelet quantity is an extra indicator for a systemic inflammatory reaction and possible microvascular thrombosis. Platelets also enables the angiogenesis facilitation *via* the cytokine VEGF, thereby promoting the growth of tumours [35]. Upon activation, the platelets also probably regulate the tumour site migration of immunocytes and haematopoietic cells, and thus facilitates the cancer-related inflammation [36]. Thirdly, as a major cellular immunity component in humans, lymphocytes are implicated in anti-cancer immunoresponses [37]. T lymphocytes, in particular, function pivotally in the tumour cell identification and killing, thereby suppressing the multiplication and motility of these cells [38]. Therefore, the reduction of lymphocytes can result in impairment of anti-tumour activity of the host.

The subgroup analysis was performed to examine the impact of treatment, cancer type, cut-off value, and study design on the results of this meta-analysis in Tables 3 and 4. The subgroup analysis indicated that sample size, study centre, country, study centre did not significantly affect the prognostic role of SII for both OS and PFS for cancer patients receiving ICIs. Additionally, SII showed obvious prognostic effect on OS and PFS for patients treated with Anti-PD-1, Anti-PD-1/PD-L1 + Anti-CTLA-4, and Anti-PD-L1 (Tables 3 and 4). Inherent heterogeneity may exist in included studies; however, subgroup analysis and publication bias test confirmed the reliability of our meta-analysis.

ICIs are applied in multiple cancer types and become standard treatment strategy for some cancers. The use of PD-1 inhibitors as adjuvant therapy in high-risk resected stage III or IV melanoma has become standard practice [39]. In the treatment of advanced gastric cancers, ICIs including nivolumab and pembrolizumab have emerged as a promising treatment option [40]. Different ICIs have been successfully introduced into clinical medicine for lung cancer treatment since 2014, including pembrolizumab, atezolizumab, and durvalumab [41]. Immune checkpoint inhibitors (ICIs) have been approved as first-line therapy for patients ineligible for cisplatin or as second-line therapy for patients with metastatic bladder cancer [42].

Recently, a series of studies also investigated SII’s prognostic value in diverse solid tumours through meta-analysis [14,43–45]. According to a meta-analysis involving 3515 patients by Zhang and colleagues, an elevated pre-therapy SII was linked significantly to worse RFS/PFS and OS among BTC patients [14]. Li et al. found significant associations of high SII with inferior PFS, cancer-specific survival (CSS) and OS among oesophageal squamous cell carcinoma (ESCC) patients receiving operation in a meta-analysis including nine retrospective studies [44]. In a meta-analysis on 10 studies enrolling 7087 patients by J. Li et al. SII displayed association with poor OS and RFS among the bladder cancer patients [45]. M. Li et al. demonstrated the connection of elevated pre-therapy SII to inferior CSS/disease-free survival (DFS)/PFS and poor OS for pancreatic cancer in their meta-analysis comprising 2132 patients [46]. In addition, a meta-analysis containing 12 studies identified the prognostic role of high SII for worse OS and PFS in patients with colorectal cancer [47]. Another recent meta-analysis with 2365 subjects reported the association between SII and short-term and long-term survival outcomes in patients with pancreatic cancer [48]. Our current meta-analysis demonstrated the significant prognostic value of SII in cancer patients treated with ICIs, which were in accordance with observations in other cancer.

The present meta-analysis has a few shortcomings. First of all, only those articles published in English were considered. Since the enrolled patients were predominantly Asians and partially Caucasians, subjects other than Caucasian and Asian ethnicities were assessed inadequately. Second shortcoming was the rather small sample size. Despite the inclusion of 17 studies, only 1990 patients were enrolled. The small sample size may also lead to the negative results regarding SII’s correlations with the clinicopathological parameters. Thirdly, the uniformed SII thresholds across the enrolled studies is a probable contributor to the meta-analytical heterogeneity. Hence, verifying our results by large-scale trials is necessary, where a unified SII threshold should be utilized.

In summary, elevated SII was linked significantly to the inferior survival prognosis (both short- and long-terms) among the ICI-receiving carcinoma patients. For these patients, SII may serve as a reliable and cheap prognostic biomarker in the clinical settings.

## Data Availability

The data that support the findings of this study are available from the corresponding author upon reasonable request.
